# The Associations Between the Anorexic Readiness Syndrome, Familism, and Body Image Among Physically Active Girls

**DOI:** 10.3389/fpsyt.2021.765276

**Published:** 2022-01-04

**Authors:** Beata Ziółkowska, Jarosław Ocalewski, Aleksandra Da̧browska

**Affiliations:** Faculty of Psychology at the Kazimierz Wielki University of Bydgoszcz, Bydgoszcz, Poland

**Keywords:** Anorexic Readiness Syndrome, familism, body image, physical activity, body weight

## Abstract

**Introduction:** *Anorexic Readiness Syndrome* (ARS) is a construct of prophylactic importance, useful in the selection of people showing a tendency to use restrictive diets and increased concentration on the body. The aim of the research was to verify the significance of the type of physical activity, body perception and familism for the development of ARS.

**Material and Method:** The research was carried out in the first half of 2021on a sample of 163 girls. It consisted of: (1) physically inactive girls (*n* = 48), (2) physically active girls in disciplines other than aesthetic (*n* = 69), (3) girls engaged in aesthetic physical activity (*n* = 46). The study used: *Anorexic Readiness Syndrome Questionnaire* (ARS-12), *Familism Scale* (FS) and *Body Image Avoidance Questionnaire* (BIAQ).

**Results:** The highest average ARS score was recorded in the group of girls engaged in aesthetic activity. A significant difference in the severity of ARS occurs between people who do not engage in activity and those who practice aesthetic activity. The severity of ARS rises as the difference between real and ideal body weight increases. People active in aesthetic disciplines who obtained a high score on the Respect scale (FS subscale) have a lower ARS score than those physically active in other disciplines who obtained low scores on the Respect scale. The higher the score on the Material success and achievement scale (FS), the greater the ARS intensity in all subgroups. What is much more important in shaping ARS is the perception of your body. The focus on eating and body weight and Clothing and appearance (BIAQ subscales) are relevant to the ARS and moderate the relationship between Material success (FS subscale) and anorexic readiness.

**Conclusions:** People engaging in aesthetic physical activity are more likely to suffer from ARS. The family can certainly prevent a child from developing anorexic readiness by shaping a sense of community and family identity, a clear division of roles, limiting the importance of materialism and competition in raising children. The prevention of ARS and eating disorders should also focus on strengthening the realistic assessment of body parameters and their acceptance, as well as promoting strategies for healthy weight control.

## Introduction

Eating disorders are increasingly becoming a public health problem worldwide (1). For example, 9% of the United States population will have an eating disorder in their lifetime (2). One of them is anorexia nervosa, which occurs in all developed and in developing countries. It is estimated that anorexia nervosa occurs in 1.4% of women and in 0.3% of men (3). From 1990 to 2017, indicators of specific eating disorders increased systematically, and they were more common in the female population than in the male population (4). In 2017, the highest rates of eating disorders were observed in Australia, Western Europe, North America–in high-income countries, but the most significant increase was recorded in East and South Asia, as well as in Guinea, Bosnia and Herzegovina and China (4–7).

According to some researchers [cf. (8, 9)], certain groups of people are particularly prone to developing symptoms of anorexia nervosa (e.g., those who practice dancing, running, skating, gymnastics), especially girls aged 15 to 19, for whom a sense of physical attractiveness has a rewarding function (10, 11).

In 2000 (12) the term *Anorexic Readiness Syndrome* (ARS) was introduced. The creation of this construct was primarily prophylactic, serving the early recognition of anorexic tendencies in children and adolescents, because the effects of dietary restrictions and the use of restrictive diets without medical or dietary control may interfere with the proper development and growth of the body (cf. 13). We assume that ARS [cf. (13, 14)] is a set of indicators located primarily in the cognitive and behavioral sphere of the functioning of an individual, suggesting abnormalities in fulfilling the nutritional needs and attitude toward one's own body and internalizing media messages regarding physical attractiveness. In other words–people who show a high level of ARS: (1) display certain behaviors and thoughts in relation to the body and food, e.g., know and follow various restrictive diets, know and use various methods of weight control; (2) have strongly internalized standards of attractiveness, and maintaining / achieving physical attractiveness is of paramount importance to them. These main features of ARS are reflected in the two-factor test tool (ARS-12) used in this study, which consists of two subscales: (1) Anorexia Sentences and Tendencies (AST) and Internalization of attractiveness norms (IAN). We also assume that the determinants of ARS are multi-faceted, similar to anorexia nervosa [cf. (15)]. Thus, among a number of internal variables important for shaping irregularities in the sphere of eating, one can indicate, i.a., interest in the corporeality and its creation, normative for adolescence, and participation in mass culture among the external ones.

While the risk factors for anorexia nervosa are very often seen in the functioning of the family system (16), the protective role of the family in revealing abnormal behavior toward the body and eating is much less frequently indicated. However, one of the determinants strengthening human mental health is familism, i.e., strong identification with the family and attachment to it (17). So far, there are few studies regarding the importance of familism for protection against the development and strengthening of eating disorders, and the available ones concern mainly enculturation processes [cf. (18, 19)].

Familism is expressed in loyalty to family members, in showing them trust, positive feelings and mutual solidarity. Its intensity is evidenced by the strength of an individual's relationship with the family, measured in relation to the strength of a person's bond with other social groups (20). According to K. Walecka-Matyja (17, p. 803), “in times referred to as anxiety, instability, unpredictability, the concept of familism takes on a special meaning, as it is indicated by researchers as one of the most important factors protecting the state of mental health.” At the same time, it seems that attention – through appropriate socialization, as well as social policy–to strengthen familism may contribute to limiting the development of mental health problems, including undertaking risky behaviors, especially in the population of children and adolescents (21, 22).

However, the matter seems much more complicated in relation to the issues related to the attitude of young people to the body and eating. As mentioned, the adolescence period is full of physiological and psychosocial changes that normatively change the perspective of young people in perceiving themselves and the world. Researchers from various regions of the world (Ukraine, Poland, the United States of America, South Africa, India) indicate that adolescents–despite different aesthetic standards in their indigenous environments–show preoccupation with a slim body and dissatisfaction with their own physicality (23–27).

Moreover, young people's involvement in popular culture, intensive use of social media, drawing inspiration from them in creating their own image, and giving physical attractiveness a superior value (28–31) make the adolescence of modern youth burdened with considerable risk. All the more so if young people are intensively engaged in physical activity, especially in the so-called aesthetic disciplines (e.g., dance, fitness) and/or those with weight categories or pressure to maintain a certain body weight. Although, on the one hand, regular physical activity may play a protective role for mental health (32), on the other hand, especially in disciplines requiring low body weight, it contributes to its deterioration, including the development of eating disorders (33–35) and the use of destructive weight control strategies (36, 37).

Attention should also be paid to gender differences in the formation and quality of the body image (38). In the case of boys, it is rather stable–it does not change significantly as a result of puberty, while in girls, along with psychophysical development, it usually changes to a disadvantage, causing a number of negative consequences (38). Cash and Pruzinsky (39) distinguish two groups of factors on which the development of body image depends. The first includes past events that cause an individual to have a specific way of thinking about oneself (e.g., the meaning given to an image by parents). The second consists of current events, especially those that force a person to pay attention to their own body and its appearance (e.g., puberty, sports activity) (40). Meanwhile, a high level of acceptance of one's body and image is desirable because it protects against distress associated with low self-esteem (41).

## Aim of the Research

The main aim of the current research was to investigate the relationship between the Anorexic Readiness Syndrome, familism, and body image among physically active girls. At the same time, it was assumed that people who are physically active, especially in disciplines related to weight categories and/or in aesthetic disciplines (e.g., dance, artistic gymnastics), may exhibit more risky eating behavior in order to control body weight. However, if they have high results of familism, the risk of ARS is lower, even with a not quite positive body image (normative in the adolescent stage). Additionally, it was predicted that the form of activity (aesthetic) or the intention to undertake it (proportions correction, reduction of fat mass for the benefit of muscle) to control physical attractiveness, correlated to a greater extent with increased values of anorexic readiness than activity aimed at increasing endurance, strength, flexibility or serving health, etc.

## Materials and Methods

The study used a set of three self-report tests and an extended demographic record. The first is the inventory of the *Anorexic Readiness Syndrome* (ARS-12) developed by Ziółkowska and Ocalewski (14). It is designed to recognize attitudes toward eating and body and the internalization of the norms of attractiveness in both girls and boys. The tool contains 12 items, to which the participant responds by answering “YES” or “NO”. Confirmatory factor analysis using the principal components method confirmed the assumptions concerning the content analysis and allowed to distinguish two factors: Anorexic sentences and tendencies (AST) and Internalization of attractiveness norms (IAN). The first of them concerns the so-called anorexic behaviors and beliefs about eating and the body, while the second–looking for inspiration in the virtual and social world to control one's attractiveness and patterns in this regard. The reliability of the tool was determined using the coefficient of internal consistency. Cronbach's alpha for the AST scale was α = 0.75, and for the IAN scale it was α = 0.80, while for the entire test it was α = 0.83 (14).

In addition, the study used *the Familism Scale* [*Mexican American Cultural Values Scales for Adolescents and Adults*, MACVS; (42)] in the Polish adaptation by K. Walecka-Matyja (17). It consists of 44 items to which the respondents refer on a scale from 1 to 5, where 1 means “I strongly disagree” and 5– “I strongly agree.” As a result of the exploratory factor analysis used in the adaptation process (17), five dimensions of familism were distinguished: (1) Respect (RESP), (2) Material success and achievements (MATSUC), (3) Individualism (IND), (4) Religion (REL), (5) Family support (FAM-SUP). The tool has satisfactory psychometric properties (the Cronbach's Alpha index value for individual subscales ranges from 0.91 to 0.63) (17).

The last of the tools used was the *Body Image Avoidance Questionnaire* (BIAQ) by Rosen et al. (43) in the Polish adaptation by Brytek-Matera and Rogoza (44). The BIAQ consists of 19 items, and the participant's task is to refer to them on a scale from 5 (“always”) to 0 (“never”). This tool is used to assess the behavioral dimension of the body image, which is related to, i.a., avoiding situations that trigger anxiety about one's own appearance (44). BIAQ has a factorial structure: (1) Clothing and appearance (CLO-APP), (2) Social activity (SOC-ACT), (3) Concentration on food and body weight (FO-WEI), (4) Preoccupation with physical appearance (PHYS-APP). In Polish studies (44), the questionnaire showed excellent internal consistency (Cronbach's alpha = 0.89), and the test-retest ratio was 0.87.

The research material was supplemented with demographics concerning the age of the respondents, the BMI, the difference in the current and ideal body weight, physical activity–type and frequency, relationships with the family, and coexisting problems in the sphere of somatic and mental health.

The research–due to the epidemic situation–was carried out using a *Google* form. The authors obtained a positive assessment of the research project of the Committee for Ethics of Scientific Research at the Faculty of Psychology of the UKW (March 16, 2019). Subsequently, an electronic version of the form was developed and the link made available to those who met the inclusion criteria (female, age 16 to 21). Additionally, the authors used the criterion of physical activity, according to which three subgroups were finally selected: (1) lack of physical activity (apart from participation in physical education lessons), (2) systematic sports activity (regular classes in a sports club / class), (3) systematic sports activity in aesthetic sports disciplines, such as classical dance, modern dance, fitness, figure skating, ballet (regular classes in a club / sports class).

The research was conducted from January 15 to February 12, 2021. After the database of results was prepared, their statistical analysis was started. The collected data was examined and descriptive statistics were calculated in line with making statistical inferences. The statistical significance of the differences between the results obtained in particular groups was verified with independent samples test ANOVA. The *r-*Person‘s correlation tests were used. Aiming to investigate the relationship between the ARS, familism and body image we conducted a series of multivariate regression to maximize the percentages of the results' variance. Mediation analysis procedure was performed according to Baron and Kenny (45). The calculations were carried out with Statistica 13 software.

## Results

One hundred and sixty-three girls aged 16 to 21 participated in the study, 48 of whom declared not being engaged in any physical activity (No activity), 46 engaged in aesthetic sports activity, and 69 physical activity in other disciplines (Sports activity). The mean age of the respondents is 18 years. The mean body mass index (BMI) is 20.84, which accounts for normal weight. The girls from the study sample, however, declared that they would like to weigh on average 3.88 kg less than their current body weight. The largest mean difference between the current and ideal body weight was recorded in the No activity group (4.34 kg). The respondents spent an average of 4.11 h a week on physical activity, undertaking this activity about three times a week (2.96). The most time for physical activity–about four times a week (3.87) was spent by girls from the Aesthetic sports activity group, which amounted to 5.86 h. About 40% of all respondents declared a burden of mental disorders other than eating disorders (e.g., anxiety, depressive disorders), about 14%–of eating disorders, and over 20%–chronic diseases (e.g., allergy, diabetes, Hashimoto's disease, cardiovascular diseases). There were no persons suffering from anorexia nervosa in the studied sample. The respondents (14%) declared symptoms of compulsive eating (it was not the same as the diagnosis of this disorder). Thirty percentage of the surveyed women come from single-parent families, and on average 62.58% indicate the problem of overweight or obesity in the family (Table 1).

**Table 1:** Characteristics of the sample.

	**Total *N = 163***	**No activity** **(1) *n* = 48**	**Sports activity (2) *n* = 69**	**Aesthetic** **sports activity (3) *n* = 46**
Age [years; *M* (*SD*)]	18.75 (1.84)	18.88 (1.86)	19.04 (1.70)	18.20 (1.91)
BMI [*kg/m^2^*; *M* (*SD*)]	20.84 (3.29)	20.75 (3.50)	21.04 (3.53)	20.62 (2.67)
Difference between current body weight and ideal body weight [*kg*; *M* (*SD*)]	−3.88 (5.69)	−4.34 (5.90)	−3.84 (6.32)	−3.44 (4.41)
Time of sport activity [*h/week; M (SD)*]	4.11 (4.11)	1.30 (1.90)	4.91 (3.82)	5.86 (4.78)
Number of active days [*no. of days/week; M (SD*)]	2.96 (1.68)	1.46 (0.92)	3.39 (1.53)	3.87 (1.48)
Mental disorders (excluding ED)	68 (41.71%)	22 (45.83%)	28 (40.58%)	18 (39.13%)
Eating disorders (ED)	23 (14.11%)	8 (16.67%)	8 (11.59%)	7 (15.22%)
Somatic diseases	37 (22.70%)	12 (25.00%)	19 (27.54%)	6 (13.04%)
Single-parent family	49 (30.06%)	17 (35.42%)	20 (28.99%)	12 (26.09%)
Occurrence of obesity/overweight in the family	102 (62.58%)	31 (64.58%)	46 (66.67%)	25 (54.35%)

*N, the size of sample; M, mean value; SD, standard deviation value*.

Next, the differences in terms of the following variables were analyzed: *Anorexic Readiness Syndrome*, familism and body image between the compared groups: 1. No activity, 2. Sports activity, 3. Aesthetic sports activity. There were statistically significant differences in the Anorexic Readiness Syndrome measured by the ARS-12 questionnaire and its AST subscale between groups 1. No activity and 3. Aesthetic sports activity (*post hoc* LSD test (Last Significant Differences): ARS SUM–*p* = 0.004 (Bonferroni correction *p* = 0.011); AST –*p* = 0.002 (Bonferroni correction *p* = 0.006). However, there were no differences between groups 1. No activity and 2. Sports activity (*post hoc* LSD test:ARS SUM–*p* = 0.294; AST–*p* = 0.132; IAN–*p* = 0.957), and also between groups 2. Sports activity and 3. Aesthetic sports activity (*post hoc* LSD test: ARS SUM–*p* = 0.033 (Bonferroni correction *p* = 0.099); AST–*p* = 0.048, Bonferroni correction *p* = 0.144.; IAN–*p* = 0.178). Generally, there were no differences in the intensity of the IAN subscale, the scale of familism and the body image (Table 2).

**Table 2:** Differences between the compared groups with regards to: Anorexia Readiness Syndrome–ARS, familism and body image.

	**Total *N = 163***	**No activity** **(1) *n* = 48**	**Sports activity (2) *n* = 69**	**Aesthetic** **sports activity (3)** ***n* = 46**	***F_**(2, 160)**_ for comparison (1–3)***
Anorexia readiness Syndrome-SUM	4.74 (2.31)	4.15 (2.13)	4.59 (2.19)	5.52 (2.49)	4.53^*^
Anorexic sentences and tendencies	2.80 (1.57)	2.33 (1.36)	2.77 (1.54)	3.35 (1.68)	5.21**
Internalization of the standards of attractiveness	1.92 (1.35)	1.81 (1.23)	1.83 (1.33)	2.17 (1.50)	1.13
Familism Scale-SUM	121.20 (20.71)	120.71 (19.07)	121.04 (21.34)	121.96 (21.80)	0.05
Respect	39.74 (10.48)	38.17 (10.01)	40.87 (10.97)	39.70 (10.21)	0.94
Material success and achievements	25.50 (6.35)	26.54 (6.81)	24.65 (5.51)	25.70 (6.96)	1.29
Individualism	20.02 (2.77)	20.54 (2.80)	19.52 (2.56)	20.22 (2.97)	2.11
Religion	14.88 (8.36)	14.92 (8.55)	14.55 (8.56)	15.32 (8.02)	0.12
Family support	25.57 (4.33)	24.96 (4.73)	26.04 (4.14)	25.50 (4.17)	0.90
Body image avoidance questionnaire					
Clothing and appearance	9.12 (2.49)	9.04 (2.66)	8.87 (2.31)	9.59 (2.56)	1.18
Social activity	2.20 (3.39)	2.19 (3.65)	2.03 (3.17)	2.46 (3.48)	0.22
Concentration on food and body weight	10.71 (4.21)	9.94 (3.58)	10.52 (3.98)	11.80 (4.97)	2.47
Preoccupation with physical appearance	10.83 (5.66)	12.06 (5.47)	10.32 (6.05)	10.30 (5.12)	1.63

*N, the size of sample*.

* *sign to indicate statistical significance*.

* p < 0.05; *p < 0.01.

The *r*-Pearson correlation analysis (Table 3) showed statistically significant relationships between ARS and all body image subscales (BIAQ): Clothing and appearance, Social activity, Concentration on food and body weight, Preoccupation with physical appearance. The greater the difference between current weight and ideal weight, the more important is the Preoccupation in body weight and physical appearance.

**Table 3:** r-Pearson correlation coefficient of the examined variables (*N* = 163).

**Variables**	**Internalization of the standards of attractive ness**	**Anorexic sentences and tendencies**	**Familism scale**	**Respect**	**Material success and achievements**	**Indivi dualism**	**Religion**	**Family support**	**Clothing and appearance**	**Social activity**	**Concentration on food and body weight**	**Preoccupation with physical appearance**
Anorexia readiness syndrome	0.76^***^	0.82^***^	0.03	0.06	0.20^*^	0.09	0.18	0.02	0.39^***^	0.38^***^	0.61^***^	0.28^***^
Internalization of the standards of attractiveness		0.25^**^	0.00	0.04	0.21^**^	0.00	0.11	0.02	0.22^**^	0.26^**^	0.39^***^	0.31^***^
Anorexic sentences and tendencies			0.04	0.06	0.11	0.13	0.16^*^	0.01	0.39^***^	0.33^***^	0.56^***^	0.15
Familism scale				0.87^***^	0.38^***^	**0.12**	0.71^***^	0.72^***^	0.01	−0.11	0.04	–0.20^*^
Respect					0.13	−0.08	0.51^***^	0.71^***^	0.07	−0.15	0.04	–0.26^**^
Material success and achievements						0.26^**^	0.07	0.00	0.23^**^	0.09	0.19^*^	0.09
Individualism							0.12	0.00	0.24^**^	0.13	0.12	0.22^**^
Religion								0.44^***^	0.21^**^	−0.12	–0.16^*^	–0.24^**^
Family support									0.03	–0.18^*^	0.06	−0.12
Clothing and appearance										0.36^***^	0.25^**^	0.47^***^
Social activity											0.39^***^	0.52^***^
Concentration on food and body weight												0.27^***^

*N, the size of sample; M, mean value; SD, standard deviation value;*

* *p < 0.05;*

** *p < 0.01;*

*** *p < 0.001*.

* *sign to indicate statistical significance*.

The analysis of empirical data shows that Internalization of the Standards of Attractiveness (IAN) correlates positively with Anorexic sentences and tendencies (AST), the Material Success and Achievement subscale (FS) and with all body image subscales (BIAQ). The significance level of these correlations was *p* < 0.01 and *p* < 0.001. Table 3 presents detailed results of the correlation between all subscales.

In the next step, multiple regression analysis was performed to check whether the subscales of the *Familism Scale* are predictors of ARS. It turned out that the subscales: 1. Respect (β^*^ = −0.24; *p* = 0.046) and 2. Material success and achievements (β^*^ = 0.24; *p* = 0.005) are statistically significant predictors of *Anorexic Readiness Syndrome*. However, the other subscales of *Familism* are not statistically significant: 3. Individualism (β^*^ = 0.04; *p* = 0.643), 4. Religion (β^*^ = −0.05; *p* = 0.586), 5. Family support (β^*^ = 0.21; *p* = 0.061). The model accounted for 9% of the ARS variance [*F*_(5,147)_ = 2.94; *p* = 0.015]. It was also checked whether the BIAQ subscales are ARS predictors. Statistically significant predictors were the subscales: 1. Clothing and appearance (β^*^ = 0.23; *p* < 0.001) and 2. Concentration on food and body weight (β^*^ = 0.51; *p* < 0.001). The subscales: 3. Social activity (β^*^ = 0.11; *p* = 0.138) and 4. Preoccupation with physical appearance (β^*^ = −0.03; *p* = 0.725) were not statistically significant. The model accounted for 42% of the ARS variance [*F*_(4.158)_ = 30.67; *p* < 0.001]. The regression analysis of multiple variables was also performed for ARS: 1. Activity time (β^*^ = 0.03; *p* = 0.724), 2. Number of active days (β^*^ = 0.33; *p* < 0.001), 3. Mental disorder (without ED) (β^*^ = 0.07; *p* = 0.394), 4. Eating disorders (ED) (β^*^ = 0.21; *p* = 0.006), 5. An incomplete family (β^*^ = 0.08; *p* = 0.252), 6. Overweight/obesity in the family (β^*^ = 0.00; *p* = 0.957), 7. Difference between the current and ideal body weight (β^*^ = 0.15; *p* = 0.042). In conclusion statistically significant predictors were: the number of days of activity, eating disorders and the difference between ideal and real weight. The model accounted for 19% of the ARS variance [*F*_(7.154)_ = 6.25; *p* < 0.001].

**Figure 1:**
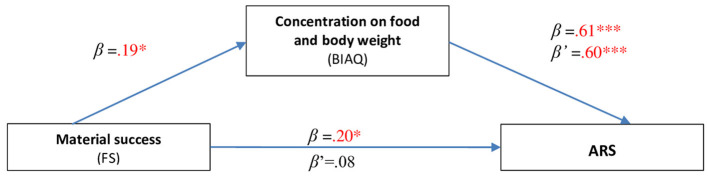
Mediating role of the factor Concentration on food and body weight (BIAQ) between Material success (FS) and ARS.

Moreover, a mediation analysis procedure was performed [according to Baron and Kenny (45)] of the BIAQ subscale Concentration on food and body weight between Material success (FS) and ARS (Figure 1).

It turned out that with an increase in the intensity of Material success, the ARS increased (β = 0.20; *p* = 0.012), and with the increase in Concentration on food and body weight (BIAQ), the ARS increased (β = 0.608; *p* < 0.001). By inserting the ARS two predictors: Material success and Concentration on food and body weight (BIAQ) into the multiple regression analysis, the variable Material success ceases to be statistically significant for ARS (β' = 0.08; *p* = 0.194). Thus, a mediating role of Preoccupation with body weight was acknowledged between material success and ARS.

**Figure 2:**
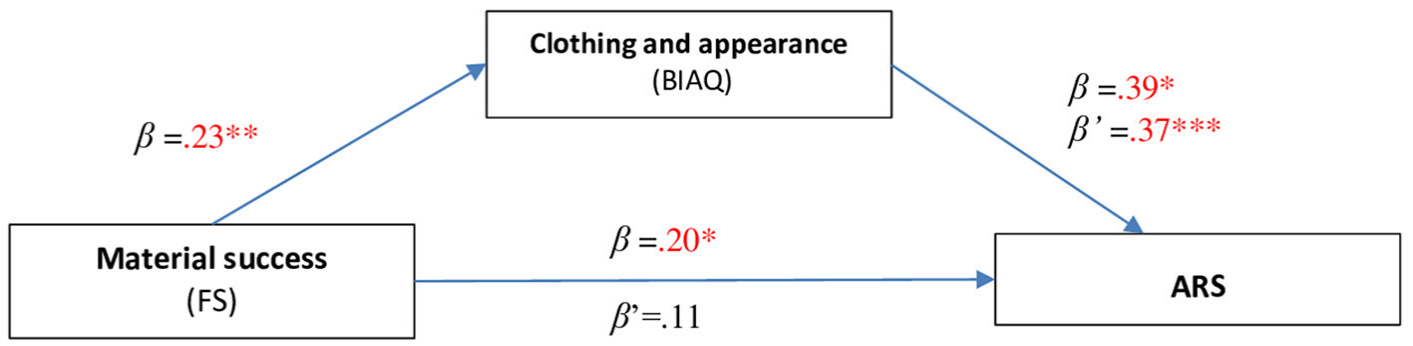
Mediating role of the factor Concentration on food and body weight (BIAQ) between Material success (FS) and ARS.

A similar statistical procedure showed a mediating role of the Clothing and appearance (BIAQ) subscale between Material success and ARS (Figure 2). By inserting the ARS two predictors: Material success and Interest in clothing (BIAQ) into the multiple regression analysis, Material success loses its statistical significance in relation to ARS (β' = 0.11; *p* = 0.133).

**Figure 3:**
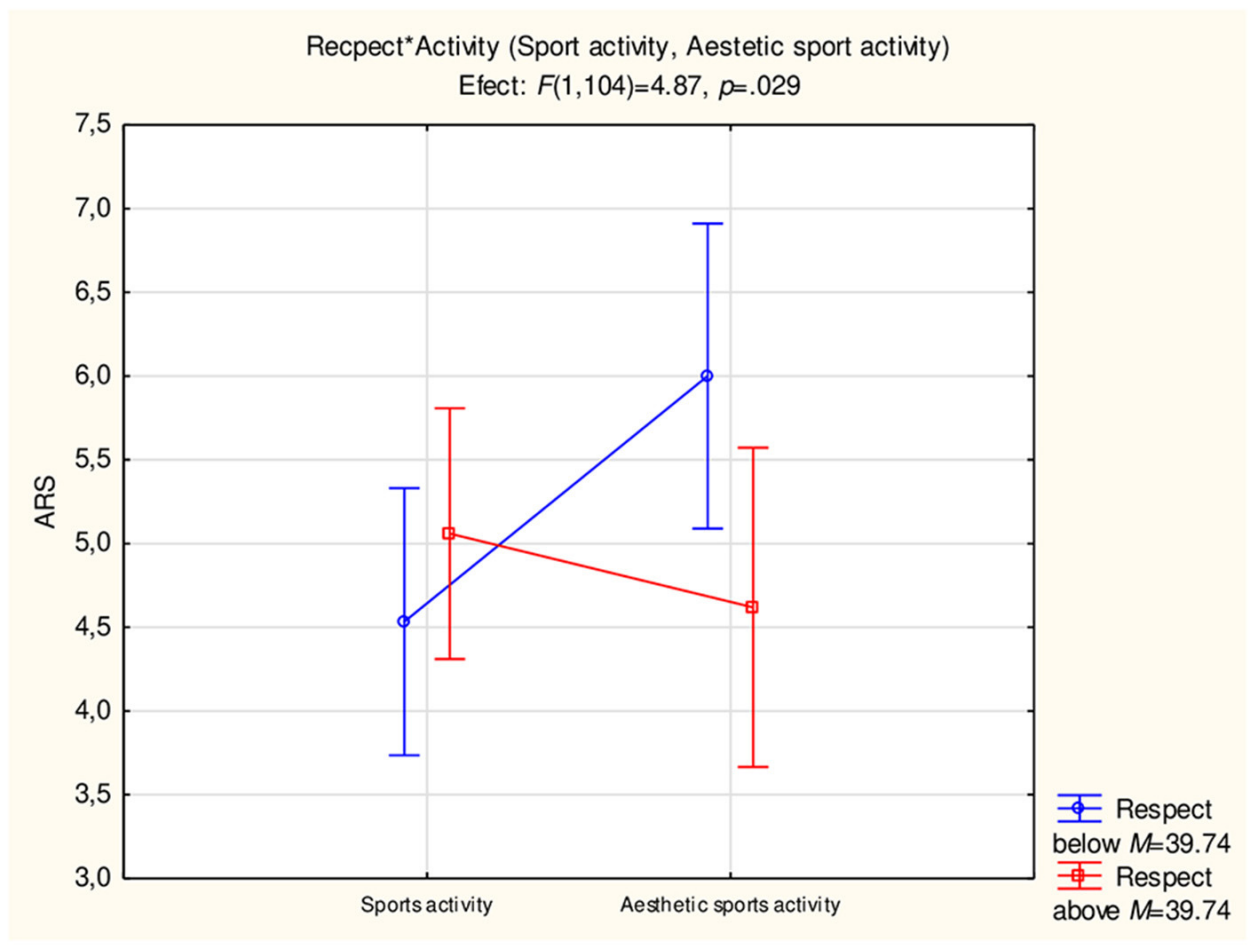
Moderating role of the factor Respect (FS) for the severity of ARS depending on sports activity.

The moderating significance of the Respect (FS) scale for the severity of ARS depending on sports activity was proved (Figure 3).

People from group three. Aesthetic sports activity, who obtained a higher score than the arithmetic mean on the Respect scale (*M* = 39.74), had a statistically significantly lower ARS score than people from the same group who obtained a lower score than the arithmetic mean on the Respect scale. On the other hand, for people from group two. Sport activity, the results, depending on the intensity of Respect, were opposite. The size of the effect of the interaction between Respect and ARS, depending on sports activity, was η^2^ = 0.04 [*F*_(1.104)_ = 4.87; *p* = 0.029].

## Discussion

The main aim of our research was: (1) to determine the intensity of ARS in the selected subgroups differing in physical activity, and (2) the relationship of the *Anorexic Readiness Syndrome* with familism and body image. It was assumed that the intensity of ARS would be significantly greater in the group of physically active people than in the group of inactive people, especially in those teenagers who practice the so-called aesthetic disciplines. Moreover, it was assumed that a higher intensity of ARS would reveal itself in people who manifest lower body satisfaction and a lower level of familism. It was expected that familism could act as a protective factor for the development and manifestation of dissatisfaction with the body by adolescents and the use of practices aimed at controlling its parameters and mass. The inspiration for this assumption were, among others, research by Sanders (46).

The obtained results indicate that the intensity of ARS measured with the ARS-12 questionnaire and its AST^1^ subscale in the studied sample is significantly different in physically inactive people and people who are active in aesthetic disciplines. In the group of people active in aesthetic disciplines, the AST subscale score is definitely higher than in physically inactive people. However, no such differences were observed between people practicing sports activities (excluding aesthetic disciplines) and physically inactive people. The AST subscale is concerned with revealing thoughts and behaviors that rely on disciplining the body, primarily to reduce its (fat) mass. Although there may be weight categories (e.g., martial arts), it seems that the importance of the figure is not as spectacular in aesthetic sports as it is in the other sports. Thus, the level of concentration on controlling the body in young people who are physically inactive and practice unsightly disciplines may be on a similar level. Moreover, the number of days of activity was a statistically significant predictor of ARS. This result is in line with the result obtained in the previous research conducted by the authors of the text (14) on a non-clinical sample of 116 people (girls−66%, boys−34%). The study included teenagers from general and sports schools. It turned out that people (interestingly–especially boys) involved in sport manifested significantly higher levels of ARS than the remaining participants.

Similar results were obtained by other researchers who verified anorexic behavior among female dancers (47). A greater severity of undesirable eating behavior was observed in this group compared to the control group. It was also shown that abnormalities in this area increase with the increase in the severity of ARS. A few years later, Chalcarz, et al. (48) documented that every fourth active young woman from the studied sample showed a high intensity of ARS, declaring the intensification of physical exercise with a simultaneous tendency to limit food-intake, as well as the use of various methods of body weight reduction and significant nutritional knowledge about the calorific value of food products. Ołpińska-Lischka (49), in a study conducted on a sample of 156 German and Polish dancers, showed that most of them were diagnosed with moderate and high levels of ARS.

Our own study did not confirm any statistically significant differences in the intensity of familism and body image between the compared groups. Meanwhile, a review of reports from studies by other authors indicates that familism is a variable that favors involvement in physical activity. Pikó and Brassai (50) investigating the relationships between various variables related to values and taking up health behaviors by young residents of Hungary and Romania found that familism, as well as religiousness and collectivism, are important for revealing pro-health (physical activity) and anti-health (use of psychoactive substances) behaviors. Ramanathan and Crocker (51) assessed the relationship between personal, family and cultural attitudes and social norms with physical activity in a study conducted among Indian teenagers. It turned out that the level of familism in the studied sample was high, and the respondents felt that physical activity was important for their physical and mental health and for strengthening their relationships with the family. However, there is data indicating the negative role of familism in maintaining improper behavior related to health. For example: McLaughlin et al. (52) found that a high level of familism, a value that reflects commitment to the family that exceeds self-care, prevents weight loss in Mexican American women. Perhaps obesity, which is a family problem, co-occurring with a high level of familism, makes it difficult to change one's eating and activity habits. This could be interpreted as betraying the family or rejecting it. However, in a situation where the family models constructive behavior, familism as integration with the family and its values seems to protect young people against non-constructive forms of functioning.

A lot of empirical data confirm significant correlations between body image and physical activity, although the results in this regard are inconclusive. For example: Haugen et al. (53) conducted research in a group of Norwegian adolescents. The authors found out that people who undertake regular physical activity are more satisfied with themselves than those who are inactive. This satisfaction was mainly related to physical appearance and body. Brytek-Matera and Kozioł (54) examined people who regularly practice fitness. The authors proved a much more positive assessment of their own body and appearance in the group of physically active women as compared to those in the control group. However, they showed that they were more likely to undertake physical activity to correct their body shape and beauty than to improve their health condition and performance, and people with high scores of self-objectification were less satisfied with their appearance. Garstka (55) has empirically documented that women practicing recreational gymnastics are more satisfied with their figure and its individual parts and endurance. However, it should be emphasized that this benefit, resulting from taking up physical activity, is not always visible, as it depends on many other variables, including motives and type of activity. When an individual is influenced by media messages and when exercise causes an obsessive focus on appearance, they become a risk factor for a person's physical and mental health, and instead of improving body satisfaction, they may reduce it (56). This is confirmed by the research of Vartanian et al. (57). The authors proved that undertaking exercise and diet motivated by body correction is associated with a greater focus on the body image than when the above-mentioned behaviors are dictated by health reasons. Vartanian et al. (57) claim that training aimed at weight loss and improving the feeling of physical attractiveness increases concentration on appearance, the risk of disturbances in the body image and engaging in behaviors harmful to the health of the individual.

Meanwhile, Schiep and Szymańska (40) empirically documented that physically active youth had a more negative body image compared to people who did not declare it. Also Hupało and Głogowska (58), while studying junior high school students, 60% of whom declared practicing physical activity, showed that adolescents are generally dissatisfied with their bodies, reveal a low sense of attractiveness, and taking up physical activity is not related to their satisfaction with their body image.

Our own research also showed statistically significant relationships between ARS and all body image subscales: Clothing and appearance, Social activity, Concentration on eating and body weight, and Preoccupation with physical appearance. In the already cited research by Ołpińska-Lischka (49) it was proved that dissatisfaction with one's own body coexists with ARS, interestingly, it concerns not so much the entire figure, but the nose, face, breasts and physical condition. Research involving girls, including those manifesting SGA (59), document that their attitude toward their own body is changed under the influence of psychocultural factors, such as, for example, the internalization of norms regarding physical attractiveness. The results coinciding with those quoted were obtained by Izydorczyk et al. (60). The authors have proven significant relationships between the self-esteem of adolescents in the period of early adolescence and selected components of the *Anorexic Readiness Syndrome*, namely dissatisfaction with the body and perception of one's own attractiveness.

Moreover, our own research has shown that the greater the difference between the real and ideal body weight, the more important for a person the preoccupation with physical appearance. This result is consistent with a number of studies [cf. (61–64)] which proved that the greater the discrepancy between the current and ideal/expected body weight by the participants, the lower their satisfaction with it. However, this relationship is probably related to gender, as girls and women are subject to socio-cultural pressures to achieve and maintain a slim figure to a greater extent than boys and men (65). As a consequence, it is the female representatives who may experience dissatisfaction with their body significantly more often/intensely, especially when its weight differs from subjective standards. Moreover, in the case of men, overall body weight is built up to a large extent by muscle mass, while in women, high body weight is usually equated with excess body fat, which results in a deterioration of the body image (66).

The presented study investigated that two subscales of familism–Respect and Material success–are significant predictors of ARS, the first of which plays the role of a destimulant, and the second–a stimulant. This means that the higher the Respect intensity, the lower the ARS intensity, and the higher the Material success intensity, the greater the ARS intensity. Due to the lack of data from other studies, it is not possible to compare them, but this result seems logical. The Respect subscale measures the need to maintain proper intergenerational relations and the importance of parents to children, e.g., in a decision-making situation. The material success subscale, on the other hand, concerns the importance of material achievements, earning money, and pursuit of achievement through competition (67). Experiencing respect in the family of origin, including the individual's physicality, may protect against initiating behaviors that are manifestations of ARS (e.g., undertaking restrictive diets, criticism of one's own attractiveness). Children growing up in families where the relations with their caregivers are correct, and they are treated by the children as significant adults, role models will be less prone to seek their attention or mark their presence by manifesting self-destructive behaviors (e.g., starvation). Moreover, the moderating role of the Respect scale for ARS severity depending on physical activity has been documented. People engaged in aesthetic activity, who obtained a higher score than the arithmetic mean on the Respect scale, showed lower ARS intensity than those with a lower score on this scale. At the same time, in the group of physically active people in other disciplines, the effect of the interaction between Respect and ARS was opposite. Probably the type of undertaken activity has a different function and allows for securing slightly different mental needs. On the other hand, materialism manifested in the family of origin, focus on objects and acquiring money, may deteriorate its functioning (68, 69), which will be felt especially by children who receive things more often than feelings and care from their caregivers.

At the same time, body image elements such as Clothing and appearance and Concentration on food and body weight were found to be predictors of ARS, with both subscales acting as stimulants for *Anorexic Readiness Syndrome*. This result is in line with other research results, including Kazmierczak et al. (70), conducted in the community of *pro-ana* people. It was found that higher ARS rates were revealed by people who were concerned about body weight, dissatisfied with their current weight regardless of its value, dissatisfied with their appearance, used food restrictions and focused on counting calories in meals. Similarly, Kozik (71), examining self-esteem as a risk factor for ARS, proved, among others, that a lower sense of physical attractiveness–an important dimension of self-assessment, predicts a higher intensity of ARS.

The authors' own research showed the mediating role of the subscales Concentration on food and body weight and Clothing and appearance (BIAQ) between Material success and ARS. This means that the higher the value of the indicated mediating variables, the higher the ARS value, regardless of the value of the Material success variable. The latter variable is associated with–as already mentioned–a greater tendency to accumulate goods, including objectification of the body by controlling its image. Material resources also favor greater availability of food on the one hand, but also weight control. Young people from families with lower economic status much more often have excess body weight and difficulties in controlling it (72).

## Conclusions

- People who are physically active in aesthetic disciplines show a greater intensity of ARS than those who are physically inactive (sports active people do not differ from no active people and aesthetic sports people in terms of ARS).
- People with a lower body image have a higher ARS intensity; additionally, as the difference between the current and ideal body weight increases, the body image deteriorates (here: Concentration on food and body weight and Preoccupation with physical appearance).
- A predictive significance for ARS is given to body image (here: the Clothing and appearance subscales and Concentration on food and body weight) and familism (here: the Respect and Material success subscales).
- The mediators between Material success (familism) and ARS are Concentration on food and body weight and Clothing and appearance (body image).
- The Respect subscale (FS) has a moderating significance for the severity of ARS depending on the type of sports activity.
- It seems that continuation of research in the group of adolescents of both sexes may reveal additional differences in ARS, body image and familism depending on the type of physical activity. Additionally, in the group of boys, it would be worthwhile to introduce the category of strength sports (e.g., bodybuilding, calisthenics, crossfit) in order to assess their risk for taking up dietary restrictions and their attitude toward their own body.

## Data Availability Statement

The raw data supporting the conclusions of this article will be made available by the authors, without undue reservation.

## Ethics Statement

The studies involving human participants were reviewed and approved by Research Ethics Committee at the Faculty of Psychology of the UKW. Written informed consent to participate in this study was provided by the participants' legal guardian/next of kin.

## Author Contributions

All authors listed have made a substantial, direct, and intellectual contribution to the work and approved it for publication.

## Conflict of Interest

The authors declare that the research was conducted in the absence of any commercial or financial relationships that could be construed as a potential conflict of interest.

## Publisher's Note

All claims expressed in this article are solely those of the authors and do not necessarily represent those of their affiliated organizations, or those of the publisher, the editors and the reviewers. Any product that may be evaluated in this article, or claim that may be made by its manufacturer, is not guaranteed or endorsed by the publisher.

## Footnotes

^1^It should be added that in the reported studies, a new tool was used to assess the ARS (11), and the results of studies by other authors concerning the assessment of *Anorexic Readiness Syndrome*, which we cite, were obtained using the *Individual Attitude to Eating Questionnaire* (12).

## References

[1] Erskine HE Whiteford HA Pike KM. The global burden of eating disorders. Curr Opin Psychiatry. (2016) 29:346–53. 10.1097/YCO.0000000000000276 27532942

[2] Deloitte Access Economics. The Social Economic Cost of Eating Disorders in the United States of America: A Report for the Strategic Training Initiative for the Prevention of Eating Disorders the Academy for Eating Disorders. (2020). Available online at: https://www.hsph.harvard.edu/striped/report-economic-costs-of-eating-disorders/ (accessed October 7, 2021).

[3] Galmiche M Déchelotte P Lambert G Tavolacci MP. Prevalence of eating disorders over the 2000–2018 period: a systematic literature review. Am J Clin Nutr. (2019) 109:1402–13. 10.1093/ajcn/nqy342 31051507

[4] Wu J Liu J Li S Ma H Wang Y. Trends in the prevalence and disability-adjusted life years of eating disorders from 1990 to 2017: results from the global burden of disease study 2017. Epidemiol Psychiatr Sci. (2020) 29:e191. 10.1017/S2045796020001055 33283690 PMC7737181

[5] Yu J Lu M Tian L Lu W Meng F Chen C. Prevalence of disordered eating attitudes among university students in Wuhu, China. Nutr Hosp. (2015) 32:1752–7. 10.3305/nh.2015.32.4.9187 26545546

[6] Nagl M Jacobi C Paul M Beesdo-Baum K Höfler M Lieb R. Prevalence, incidence, and natural course of anorexia and bulimia nervosa among adolescents and young adults. Eur Child Adolesc Psychiatry. (2016) 25:903–18. 10.1007/s00787-015-0808-z 26754944

[7] Pegado P Alckmin-Carvalho F Leme D Carneiro F Kypriotis P Camacho P. Development, applicability and effects of a pilot program of group cognitive-behavioral therapy in Brazilian adolescents with anorexia nervosa. Arch Clin Psychiatry. (2018) 45:57–60. 10.1590/0101-60830000000158

[8] Mehler PS Brown C. Anorexia nervosa-medical complications. J Eat Disord. (2015) 3:11. 10.1186/s40337-015-0040-8 25834735 PMC4381361

[9] Paixão C Dias CM Jorge R Carraça EV Yannakoulia M de Zwaan M. Successful weight loss maintenance: a systematic review of weight control registries. Obes Rev. (2020) 21:e13003. 10.1111/obr.13003 32048787 PMC9105823

[10] Smink FR van Hoeken D Hoek HW. Epidemiology of eating disorders: incidence, prevalence and mortality rates. Curr Psychiatry Rep. (2012) 14:406–14. 10.1007/s11920-012-0282-y 22644309 PMC3409365

[11] Wagner G Zeiler M Waldherr K Philipp J Truttmann S Dür W. Mental health problems in Austrian adolescents: a nationwide, two-stage epidemiological study applying DSM-5 criteria. Eur Child Adolesc Psychiatry. (2017) 26:1483–99. 10.1007/s00787-017-0999-6 28540609 PMC5701961

[12] Ziółkowska B. Uwarunkowania ekspresji syndromu gotowości anorektycznej [Polish: Conditions for the expression of the anorexic readiness syndrome], Sb Prace Filozoficke Fakulty Brnenske Univerzity Studia Minora Facultatis Philosophicae Universitastis Brunensis P4, (2000).

[13] Kulkarni AA Swinburn BA Utter J. Associations between diet quality and mental health in socially disadvantaged New Zealand adolescents. Eur J Clin Nutr. (2015) 69:79–83. 10.1038/ejcn.2014.130 25028085

[14] Ziółkowska B Ocalewski J. Anorexia Readiness Syndrome–about the need for early detection of dietary restrictions. Pilot study findings. Psychiatr Pol. (2021) 55:1079–91. 10.12740/PP/120411 34997744

[15] Le Grange D Hughes EK Court A Yeo M Crosby RD Sawyer SM. Randomized clinical trial of parent-focused treatment and family-based treatment for adolescent anorexia nervosa. J Am Acad Child Adolesc Psychiatry. (2016) 55:683–92. 10.1016/j.jaac.2016.05.007 27453082

[16] Mensi MM Rogantini C Nacinovich R Riva A Provenzi L Chiappedi M. Clinical features of adolescents diagnosed with eating disorders and at risk for psychosis. Eur Psychiatry. (2020) 63:e80. 10.1192/j.eurpsy.2020.80 32829729 PMC7503175

[17] Walecka-Matyja KK. Familizm–pojecie, pomiar i znaczenie dla zdrowia psychicznego. [Polish: Familism - concept, measurement and significance for mental health]. Psychiatr Pol. (2020) 54:791–806. 10.12740/PP/108993 33386728

[18] Austin SB Ziyadeh NJ Forman S Prokop LA Keliher A Jacobs D. Screening high school students for eating disorders: results of a national initiative. Prev Chronic Dis. (2008) 5:A114. 18793502 PMC2578782

[19] Bettendorf SK Fischer AR. Cultural strengths as moderators of the relationship between acculturation to the mainstream US Society and eating- and body-related concerns among Mexican American women. J Couns Psychol. (2009) 56:430–40. 10.1037/a0016382

[20] Ignatowski G. “Familizm i nepotyzm. Rodzina w firmach rodzinnych”. [Polish: Familism and nepotism. Family in family businesses] In: Piasecki B, Marjański A, editors. Firmy rodzinne–wyzwania współczesności. Przedsiebiorczość i Zarzadzanie [Polish: Family businesses-contemporary challenges. Entrepreneurship and Management], XVII(6), III, Społeczna Akademia Nauk, Łódz. (2016). p. 181–91.

[21] Zeiders KH Umaña-Taylor AJ Derlan CL. Trajectories of depressive symptoms and self-esteem in Latino youths: examining the role of gender and perceived discrimination. Dev Psychol. (2013) 49:951–63. 10.1037/a0028866 22686175

[22] Stein GL Gonzalez LM Cupito AM Kiang L Supple AJ. The Protective Role of Familism in the Lives of Latino Adolescents. J Fam Issues. (2015) 36:1255–73. 10.1177/0192513X13502480

[23] McKnight Investigators. Risk factors for the onset of eating disorders in adolescent girls: results of the McKnight longitudinal risk factor study. Am J Psychiatry. (2003) 160:248–54. 10.1176/ajp.160.2.248 12562570

[24] Tiunova A. Relationship of body image and self-esteem in adolescents with different types of constitutional development: preliminary results. Act Nerv Super. (2015) 57:81–6. 10.1007/BF03379626

[25] Pedro TM Micklesfield LK Kahn K Tollman SM Pettifor JM Norris SA. Body image satisfaction, eating attitudes and perceptions of female body silhouettes in rural south african adolescents. PLoS ONE. (2016) 11:e0154784. 10.1371/journal.pone.0154784 27171420 PMC4865095

[26] Mannat MS Shradha SP Bhumika TV. Body image, eating disorders and role of media among Indian adolescents. J Indian Assoc Child Adolesc Ment Health. (2016) 12:9–35. Available online at: https://www.jiacam.org/1201/orig1jan2016.pdf

[27] Wojtyła-Buciora P Klimberg A Wojtyła A. Samoocena własnej sylwetki a wskaznik masy ciała młodziezy w Polsce. [Polish: Self-esteem of one's own figure and the body mass index of adolescents in Poland] *Probl Hig Epidemiol*. (2018) 99:146–54. Available online at: http://www.phie.pl/pdf/phe-2018/phe-2018-2-146.pdf

[28] Cohen R Blaszczynski A. Comparative effects of Facebook and conventional media on body image dissatisfaction. J Eat Disord. (2015) 3:23. 10.1186/s40337-015-0061-3 26140215 PMC4489037

[29] Turner PG Lefevre CE. Instagram use is linked to increased symptoms of orthorexia nervosa. Eat Weight Disord. (2017) 22:277–84. 10.1007/s40519-017-0364-2 28251592 PMC5440477

[30] Tiggemann M Slater A. Facebook and body image concern in adolescent girls: a prospective study. Int J Eat Disord. (2017) 50:80–3. 10.1002/eat.22640 27753130

[31] Steinsbekk S Wichstrøm L Stenseng F Nesi J Hygen BW Skalicka V. The impact of social media use on appearance self-esteem from childhood to adolescence–A 3-wave community study. Comput Hum Behav. (2020) 114. 10.1016/j.chb.2020.106528

[32] Meyer C Taranis L Goodwin H Haycraft E. Compulsive exercise and eating disorders. Eur Eat Disord Rev. (2011) 19:174–89. 10.1002/erv.1122 21584911

[33] Anderson L Reilly E Gorrell S Schaumberg K. AndersonD. Gender-based differential item function for the difficulties in emotion regulation scale. Pers Individ Differ. (2016) 92:87–91. 10.1016/j.paid.2015.12.016

[34] McLester CN Hardin R Hoppe S. Susceptibility to eating disorders among collegiate female student-athletes. J Athl Train. (2014) 49:406–10. 10.4085/1062-6050-49.2.16 24762233 PMC4080598

[35] Quatromoni PA. A tale of two runners: a case report of athletes' experiences with eating disorders in college. J Acad Nutr Diet. (2017) 117:21–31. 10.1016/j.jand.2016.09.032 28010854

[36] Filaire E Maso F Degoutte F Jouanel P Lac G. Food restriction, performance, psychological state and lipid values in judo athletes. Int J Sports Med. (2001) 22:454–9. 10.1055/s-2001-16244 11531040

[37] Martínez Rodríguez A Vicente Salar N Montero Carretero C Cervelló Gimeno E Roche Collado E. Eating disorders and diet management in contact sports; EAT-26 questionnaire does not seem appropriate to evaluate eating disorders in sports. Nutr Hosp. (2015) 32:1708–14. 10.3305/nh.2015.32.4.9214 26545540

[38] Biernat M Bak-Sosnowska M. Postawa ciała a obraz siebie i funkcjonowanie psychospołeczne w okresie adolescencji. [Polish: Body posture regarding self-image and psychosocial functioning in adolescence] *Pediatria i Medycyna Rodzinna*. (2018) 14:282–5. 10.15557/PiMR.2018.0031

[39] Cash TF Pruzinsky T. “Future challenges for body image theory, research, and clinical, practice”. In Cash TF, Pruzinsky T., editors. Body Images: A Handbook of Theory, Research, and Clinical Practice. New York: Guilford Press (2002). p. 509-516.

[40] Schiep S Szymańska P. Obraz ciała, stres i samoregulacja u osób uprawiajacych fitness w wieku póznej adolescencji i wczesnej dorosłości. [Polish: Body image, stress and self-regulation in late adolescence and early adult fitness practitioners] *Psychologia Rozwojowa*. (2012) 17:97–113. 10.4467/20843879PR.12.021.0640

[41] Brandt-Salmeri A Przybyła-Basista H. Body image and self-esteem in women with breast cancer–the role of body acceptance. Psychoonkologia. (2018) 22:1–10. 10.5114/pson.2018.81160

[42] Knight GP Gonzales NA Saenz DS Bonds DD Germán M Deardorff J. The Mexican American cultural values scales for adolescents and adults. J Early Adolesc. (2010) 30:444–81. 10.1177/0272431609338178 20644653 PMC2904976

[43] Rosen JC Srebnik D Saltzberg E Wendt S. Development of a body image avoidance questionnaire. Psychol Assess. (1991) 3:32–7. 10.1037/1040-3590.3.1.32

[44] Brytek-Matera A Rogoza R. The polish version of the body image avoidance questionnaire: an exploratory structural equation modeling approach. Eat Weight Disord. (2016) 21:65–72. 10.1007/s40519-015-0206-z 26183601

[45] Baron RM Kenny DA. The moderator-mediator variable distinction in social psychological research: conceptual, strategic, and statistical considerations. J Pers Soc Psychol. (1986) 51:1173–82. 10.1037/0022-3514.51.6.1173 3806354

[46] Sanders S. Skin Tone, Body Image and Familismo: an Investigation of Latina Women. Ohio: The University of Akron (2019).

[47] Chalcarz W Musieł A Koniuszuk K. Ocena zachowań anorektycznych tancerek w zalezności od poziomu syndromu gotowości anorektycznej. Pol J of Sports Med. (2008) 1:21–9.

[48] Chalcarz W Merkiel S Milewska J. Ocena zachowań anorektycznych wśród studentek turystyki i rekreacji. [Polish: Assessment of anorexic behavior of dancers depending on the level of anorexic readiness syndrome]. Probl Hig Epidemiol. (2014) 95:310–6. Available online at: http://www.phie.pl/pdf/phe-2014/phe-2014-2-310.pdf

[49] Ołpińska-Lischka M. Assessment of anorexia readiness syndrome and body image in female dancers from Poland and Germany. J Educ Health Sport. (2017) 7:423–40. Available online at: 10.5281/zenodo.834093

[50] Pikó B Brassai L. Values and health-related behavior. Eur J Ment Health. (2007) 2:171–81. 10.1556/EJMH.2.2007.2.3

[51] Ramanathan S Crocker PR. The influence of family and culture on physical activity among female adolescents from the Indian diaspora. Qual Health Res. (2009) 19:492–503. 10.1177/1049732309332651 19299755

[52] McLaughlin EA Campos-Melady M Smith JE Serier KN Belon KE Simmons JD. The role of familism in weight loss treatment for Mexican American women. J Health Psychol. (2017) 22:1510–23. 10.1177/1359105316630134 26929169

[53] Haugen T Säfvenbom R Ommundsen Y. Physical activity and global self-worth: the role of physical self-esteem indices and gender. Ment Health Phys Act. (2011) 4:49–56. 10.1016/j.mhpa.2011.07.001

[54] Brytek-Matera A Kozioł A. Zaburzenia odzywiania. [Polish: Eating disorders.] Warszawa: PZWL (2015).

[55] Garstka K. Analiza zmian w percepcji i ocenie obrazu własnego ciała u kobiet w średnim wieku uprawiajacych gimnastyke rekreacyjna. [Polish: Analysis of changes in the perception and assessment of body image in middle-aged women practicing recreational gymnastics.] Nowa Medycyna. (2000) 12:97–8. Available online at: http://www.czytelniamedyczna.pl/1612,analiza-zmian-w-percepcji-i-ocenie-obrazu-wasnego-ciaa-u-kobiet-w-rednim-wieku-u.html

[56] Guszkowska M. Aktywność fizyczna i psychika–korzyści i zagrozenia. [Polish: Physical activity and psychic-benefits and threats.] Toruń: Wyd. Adam Marszałek (2013).

[57] Vartanian L. Wharton Ch, Green E. Appearance vs health motives for exercise and for weight loss. Psychol Sport Exerc. (2012) 13:251–6. 10.1016/j.psychsport.2011.12.005

[58] Hupało A Głowska J. Wizerunek ciała aktywnej i nieaktywnej fizycznie młodziezy gimnazjalnej ZSP w Goszczynie. [Polish: Image of the body of physically active and inactive junior high school students from ZSP (TN: public school) in Goszczyna] *Zeszyty Naukowe WSKFiT*. (2016) 11:55–60. Available online at: https://www.wskfit.pl/PDF/artykuly/16/16036-hupalo.pdf

[59] Sciegienny A. Syndrom gotowości anorektycznej i percepcja wiezi rodzicielskiej a postawa wobec własnego ciała u dziewczat w okresie adolescencji. [Polish: Anorexic readiness syndrome and perception of parental bond and attitude towards one's own body in adolescent girls.] Kraków: UJ (2019).

[60] Izydorczyk B Gut A Dobrowolska M. Samoocena i wpływ socjokulturowy na wizerunek ciała a gotowość do zachowań anorektycznych. [Polish: Self-esteem and socio-cultural influence on the body image and readiness for anorexic behavior.] Czasopismo Psychologiczne (2018) 1:25–226. 10.14691/CPPJ.24.1.213

[61] Bessenoff G. Can the media affect us? social comparison, self-discrepancy, and the thin ideal. Psychol Women Q. (2006) 30:239–51. 10.1111/j.1471-6402.2006.00292.x

[62] Bratovcic V Mikic B Kostovski Z Teskeredzic A Tanovic I. Relations between different dimensions of self-perception, self-esteem and body mass index of female students. Int J Morphol. (2015) 33:1338–42. 10.4067/S0717-95022015000400024

[63] Zarychta K Chan CKY Kruk M Luszczynska A. Gender-specific body areas satisfaction and body weight status in adolescents: mediating effects of physical activity, fruit and vegetable intake, and energy-dense food intake. Appl Psychol Health Well Being. (2019) 11:80–101. 10.1111/aphw.12145 30288920 PMC7379245

[64] Zarychta K Horodyska K Chan CKY. Body areas satisfaction and body mass in adolescents: mediating effects of actual-ideal body weight discrepancies. Eat Weight Disord. (2020) 25:1011–9. 10.1007/s40519-019-00722-8 31175619 PMC7399669

[65] Striegel-Moore R Bulik C. Risk factors for eating disorders. Am Psychol. (2007) 62:181–98. 10.1037/0003-066X.62.3.181 17469897

[66] Heinberg LJ Coughlin JW Pinto AM Haug N Brode C Guarda AS. Validation and predictive utility of the sociocultural attitudes toward appearance questionnaire for eating disorders (SATAQ-ED): internalization of sociocultural ideals predicts weight gain. Body Image. (2008) 5:279–90. 10.1016/j.bodyim.2008.02.001 18640081

[67] Walecka-Matyja K. Familizm a orientacja wspólnotowa i materializm w okresie dorosłości. [Polish: Familism and community orientation and materialism in adulthood] *Kwartalnik Naukowy Fides et Ratio*. (2020) 1:241–59. 10.34766/fetr.v41i1.231

[68] Górnik-Durose M. Nowe oblicze materializmu czyli z deszczu pod rynne. [Polish: The new face of materialism, i.e. from the frying pan into the fire.] *Psychologia. Edukacja i Społeczeństwo*. (2007) 211–26. Available online at: ttps://www.kul.pl/files/714/przeglad_psychologiczny_2020_nr_2.pdf

[69] Poraj-Weder M. Niedaleko pada jabłko od jabłoni? O transmisji materialistycznych wzorców w diadzie matka-córka. [Polish: A chip off the old block. On the transmission of materialistic patterns in the mother-daughter dyad.] Bydgoszcz: Wydawnictwo Uniwersytetu Kazimierza Wielkiego w Bydgoszczy (2017).

[70] Kazmierczak N Kiełbasa S Patryn R Niedzielski A. Zachowania anorektyczne wśród społeczności pro-ana. [Polish: Anorexic behaviors among the pro-ana community.] *Med Ogólna Nauki Zdr*. (2015) 21:168–73. 10.5604/20834543.1152915

[71] Kozik P. Samoocena i jej wymiary a ekspresja syndromu gotowości anorektycznej wśród dziewczat (praca magisterska) [Polish: Self-esteem and its dimensions and the expression of the anorexic readiness syndrome among girls (master's thesis)] Kraków: Repozytorium Uniwersytetu Jagiellońskiego (2018).

[72] Tabak I Jodkowska M Oblacińska A Mikiel-Kostyra K. Czy spozywanie wspólnych posiłków z rodzina moze chronić nastolatki przed otyłościa? [Polish: Can eating food together with the family protect peens from obesity?] *Dev Period Med*. (2012) XVI:313–22. 23378411

